# Anxiolytic activity of *Nymphaea alba* Linn. in mice as experimental models of anxiety

**DOI:** 10.4103/0253-7613.75670

**Published:** 2011-02

**Authors:** B.S. Thippeswamy, Brijesh Mishra, V.P. Veerapur, Gourav Gupta

**Affiliations:** Department of Pharmacology, Sree Siddaganga College of Pharmacy, Tumkur - 572 102, Karnataka, India; 1 Quality Assurance, Sree Siddaganga College of Pharmacy, Tumkur - 572 102, Karnataka, India

**Keywords:** Anxiolytic, elevated plus maze, exploratory, foot-shock, *Nymphaea alba*

## Abstract

**Objective:**

The aim of the present work was to evaluate the anxiolytic effect of an ethanolic extract of *Nymphaea alba* Linn. in mice.

**Materials and Methods:**

The elevated plus maze test (EPMT), light and dark test (L and DT) and open field test (OFT) were used to assess the anxiolytic activity of the ethanolic extract of *N. alba* Linn. in mice. In addition, aggressive behavior and motor coordination was also assessed by foot shock induced aggression test (FSIAT) and rota rod test (RRT). Diazepam 1 mg/kg served as a standard anxiolytic drug, administered orally.

**Results:**

The ethanolic extract of *N. alba* (100 and 200 mg/kg, p.o.) significantly increased the percentage of time spent and number of entries in open arm in EPMT. In L and DT, the extract produced significant increase in time spent, number of crossing and decrease in the duration of immobility in light box. In OFT, the extract showed significant increase in number of rearings, assisted rearings and number of square crossed, all of which are demonstrations of exploratory behavior. In FSIAT, *N. alba* extract attenuated aggressive behavior related to anxiolytic activity, such as number of vocalization, leaps, rearing, biting/attacks and facing each other in paired mice. Furthermore, the extract produced skeletal muscle relaxant effect assessed by RRT.

**Conclusion:**

The results of the present study suggest that an ethanolic extract of *N. alba* may possess anxiolytic activity and provide a scientific evidence for its traditional claim.

## Introduction

Anxiety disorder is increasingly recognized as a highly prevalent and chronic disorder with onset during the teenage years, with an incidence of 18.1% and a lifetime prevalence of 28.8%.[1] The disorder is associated with significant disability (including educational and occupational) which has a negative impact on the quality of life.[2] Pharmacotherapeutic approaches for the management of anxiety disorders include psychotropic drugs, but these agents are limited by their side-effect profile, the need for dietary precautions, and drug interactions.[3] Regular use of benzodiazepines causes deterioration of cognitive functioning, addiction, psychomotor impairment, confusion, aggression, excitement, anterograde amnesia, physical dependence, and tolerance.[4] These are some of the factors that caused interest in many researchers to evaluate new compounds from plant origin in the hope to identifying other anxiolytic drugs with fewer unwanted side effects.

Various types of herbal medicines have been used as anxiolytic agents in different part of the world, such as *Citrus aurantium* from Brazil-Indians, Afro-Brazilians and Caboclos,[5] roots of kava plant from the topical pacific region, and the saponin-containing fraction of leaves of *Albizia lebbeck* from India are all known to have anxiolytic effects.[6] The major obstruction in the application of herbal medicine into medical practice is the lack of sufficient scientific and clinical data and better understanding of efficacy and safety of the herbal products.

*Nymphaea alba* Linnaeus (family Nymphaeaceae), commonly known as white water lily in English and kumuda in Sanskrit, is an aquatic herb with perennial rhizomes or rootstocks anchored with mud. It is globally distributed in Europe, North Africa, Southwest Asia, India, China and Russia. It is rich in tannic acid, gallic acid, alkaloids, sterols, flavonoids, glycosides, hydrolyzable tannins and high–molecular-weight polyphenolic compounds.[5] All the parts of the plant have medicinal uses in traditional system of medicine. It is used as an aphrodisiac, anodyne, antiscrophulatic, astringent, cardiotonic, demulcent, sedative and anti-inflammatory. Further, it also produces calming and sedative effects upon the nervous system, and is useful in the treatment of insomnia, anxiety and similar disorders.[7–9] Its anticarcinogenic action and inhibition of renal oxidative stress and hyperproliferative response were reported.[10,11] However, so far, its effect on CNS activity has not been studied. Therefore, we undertook the study to evaluate the anxiolytic potential of *N. alba*, by using different animal models and studying the effect of the plant on their exploratory behavior.

## Materials and Methods

### 

#### Plant Material

The entire plant was collected from Siddrabhetta, National Botanical Garden, Tumkur, Karnataka, during August 2009. The plant was identified and authenticated by Dr. Siddappa, Prof. and Head of the Department of Botany, Sree Siddaganga College of Arts, Science and Commerce for Boys, B.H. Road, Tumkur. A voucher specimen is preserved in our laboratories.

#### Preparation of Extract

The entire plant material was thoroughly washed with water, dried under shade condition and coarsely powdered. The powder (750 g) was extracted with 95% distilled ethanol by using Soxhlet Extractor apparatus for 18 hours. The extract was concentrated to a small volume and evaporated to dryness in vacuum desiccators. The yield of ethanolic extract of *N. alba* was found to be 11% w/w. The extract was stored in a refrigerator and used for the pharmacological investigations.

#### Drugs and Chemicals

Diazepam tablets (Calmpose ^®^ 5 mg, Ranbaxy Laboratories Limited Navi-Mumbai, India B-No. 1700593) were used as a standard drug. Diazepam was suspended in 0.5% of carboxymethyl cellulose in distilled water. Two different doses (100 and 200 mg/kg) of *N. alba* extract were prepared by dissolving with 1% of gum acacia in distilled water. All solutions were prepared freshly on the test days and administered orally (p.o.).

#### Animals

Male Swiss albino mice (22–25 g) were used in the study. This was done in order to avoid the influence of ovarian hormone fluctuations across the estrous cycle in female mice. The behavioral observations took place in sound proof rooms at the same period of the day to reduce the confounding influence of diurnal variation in spontaneous behavior. The Institutional Animal Ethics Committee (IAEC) approved the experimental protocol (SSCPT/IAEC/58/2008-09) dated 17/11/2008. All procedures were performed in accordance with IAEC, constituted as per the direction of the Committee for the Purpose of Control and Supervision of Experiments on Animals (CPCSEA), Government of India, New Delhi. All the animals were obtained from the animal house of Sree Siddaganga College of Pharmacy, Tumkur, Karnataka, where they were housed in groups of six mice per cages and maintained under standard environmental conditions: 25 ± 2°C temperature, 12:12 hour light and dark cycle, and 45–55% relative humidity, with free access of food and water *ad libitum*. Food, not water, was withdrawn 6 hours before and during the experiment. All the experiments were carried out during the light period (0800:1600 hours).

#### Acute Oral Toxicity Studies

Acute oral toxicity test was performed according to Organization for Economic Co-operation and Development (OECD) guideline test, ANNEX-423. Ethanolic extract of *N. alba* at different doses 5, 50, 300 and 2000 mg/kg was administered orally to female mice. The literature search of conventional LD_50_ tests shows that usually there is little difference in sensitivity between the sexes, but in those cases where differences are observed, females are generally slightly more sensitive than males. This was the reason behind choosing female mice for the toxicity studies.[12] The animals were observed for signs of toxicity such as hyperactivity, grooming, convulsions, sedation, hypothermia continuously for 2 hours, and for mortality up to 24 hours, after administration of the doses.

#### Behavioral Assessment of Anxiolytic Activity

#### Treatment schedule

The anxiolytic activity was examined by using the elevated plus maze (EPM) test, open field test (OFT), light and dark test (L and DT), foot shock induced aggression test (FSIAT) and motor coordination test assessed by rota rod test (RRT). The animals were divided into four groups, with each group consisting of six male mice. Group 1 received vehicle (normal saline); group 2 received diazepam (1 mg/kg); groups 3 and 4 received *N. alba* extract (100 and 200 mg/kg).

#### Elevated plus maze test

The EPMT apparatus consisted of four arms elevated 30 cm above the floor, with each arm positioned at 90° relative to the adjacent arms. Two of the arms were enclosed with high walls (30 × 7 × 20 cm), and the other arms were connected via a central area (7 × 7 cm) to form a plus sign. The maze floor and the walls of enclosed arms were painted black. The room was illuminated with a 40-W lamp at the central platform. The animals were treated with vehicle, extract and diazepam orally, 60 min prior to the test. The experiment was performed between 0900 and 1400 hours, and the mice became accustomed to the dimly lit experimental laboratory for 30 min prior to behavioral testing. Each mouse was individually placed on the central platform facing toward an open arm. The frequency and duration of entries into the open and closed arms were observed for 5 min. An entry was counted when all four paws of the mouse entered an open or closed arm. Subsequently, the percentage of time spent (duration) in the open arms [100 × open/(open + enclosed)] and percentage of the number of open arm entries (frequency, 100 × open/total entries) were calculated for each animal. The apparatus was thoroughly cleaned after each trial.[13]

#### Open field test

The apparatus consisted of a wooden box (60 × 60 × 60 cm). The arena of the open field was divided into 16 squares (15 × 15 cm): the four inner squares in the center and 12 squares in the periphery along the walls. The experimental room was a sound attenuated, dark room. The open field arena was illuminated with a 40-W lamp, focusing on the field from a height of about 75–100 cm. After 60 min of oral treatment with vehicle, diazepam (1 mg/kg) and *N. alba* extract (100 and 200 mg/kg), animals were placed individually in one of the corner squares and number of rearings, assisted rearings and number of squares crossed were observed for 5 min.[14]

#### Light and dark test

The L and DT apparatus consisted of open top wooden box. Two distinct chambers, a black chamber (25 cm long × 35 cm wide × 35 cm deep), painted black and made dark by covering its top with black plywood, and a bright chamber (25 cm long × 35 cm wide × 35 cm deep), painted white and brightly illuminated with 40-W white light source, were placed 25 cm above the open box. The two chambers were connected through a small open doorway, (7.5 cm long × 5 cm wide) situated on the floor level at the center of the partition. The mice were placed individually in center of the light box after 60 min of oral treatments and observed for 5 min.[15]

#### Foot shock induced aggression test

A pairs of mice was placed in the aggressometer (Medicraft aggressometer, INCO, Instruments & Chemicals (P) LTD. Model Town, Ambala City, India, model no. 514/1) which consisted of steel rods at the bottom with a diameter of 6 mm and is enclosed within a Perspex chamber for clear visibility of the animals. A 60-Hz current was delivered to the foot of the animal for a total of 3 min. A period of relaxation of 5 sec was provided between two successive shocks of 5 sec during the 3-min recording. After 60 min of the oral treatments, the animals were observed for 3 min for i) vocalization ii) leaps iii) biting/attacks iv) rearing and v) facing each other.[16]

#### Motor coordination test by rota rod

Rota rod apparatus consisted of a base platform and an iron rod of 3 cm diameter and 30 cm length, with a non-slippery surface. This rod was divided into three equal sections by two disks, thus enabling three mice to walk on the rod at the same time at the speed of 32 rpm. Intervals between the mounting of the animal on the rod and falling off of it were recorded as the performance time. The training of mice in RRT was given 20 times at 5–15 min intervals. Thereafter, three mice were randomly selected to determine the retention of the walking technique. Other animals that performed on the rod for more than 10 sec were used for assaying the drug effects. The remaining animals showing poor results or the ones that did not reach the criterion (10 sec) were excluded from the experiments. After the administration of the standard drug and extract, the performance time was measured at 15-min time intervals for 90 min for 300 sec (cut-off time). For the assay of each dose, 24 trained mice were used, and repetitions of the drug test were never made with the same animal.[17]

#### Statistical Analysis

All the results were expressed as mean ± SEM and the data were analyzed using one-way analysis of variance (ANOVA) followed by Dunnett’s “*t*” test. A *P* value of <0.05 was considered as the level of significance.

## Results

### 

#### Acute Oral Toxicity Study

The ethanolic extract of *N. alba* was orally administered to different groups of mice (three mice per group) at doses of 5, 50, 300 and 2000 mg/kg body weight, respectively. The animals were observed for 48 hours to study their general behavior and to detect signs of discomfort and nervous manifestations. As even the mice receiving the highest dose of *N. alba* (2000 mg/kg body weight, p.o.) did not show any mortality, dose levels at 1/10^th^ (200 mg/kg body weight, p.o.) and 1/20^th^ (100 mg/kg body weight, p.o.) of this highest dose were selected for the anxiolytic activity.

#### Elevated Plus Maze

Diazepam treated mice showed significant increase (*P* < 0.05) in the number of open arm entries, time spent in open arms and the number of rears in the open arm. They showed a reduction in the time spent in closed arm [Table 1]. *N. alba* extract treated mice exhibited significant increase (*P* < 0.05) in the number of open arm entries (100 and 200 mg/kg), time spent in open arm, percentile ratio of open arm to total arm entries, the number of total arm entries, and the number of rears in the open arm entries, but decrease in time spent in closed arm [Table 1].

**Table 1:** Effect of *N. alba* extract on behavior of mice in elevated plus maze test

*Treatment (mg/kg, p.o.)*	*No. of entries*		*Time spent (sec)*		*% OAE%*	*TSOA (sec)*
	*Open arm*		*Closed arm*		*Open arm*	*Closed arm*
Vehicle	5.00 ± 0.57	19.00 ± 1.41	32.67 ± 6.67	199.2 ± 13.74	21.00 ± 2.74	14.27 ± 3.22
Diazepam (1)	10.50 ± 1.08^b^	12.33 ± 0.88^b^	65.67 ± 7.34^a^	107.0 ± 5.75^c^	45.61 ± 2.62^c^	37.73 ± 3.13^c^
*N. alba* extract (100)	9.83 ± 1.24^a^	10.83 ± 1.01^c^	64.67 ± 5.83^a^	91.67 ± 8.97^c^	46.91 ± 2.40^c^	41.39 ± 1.48^c^
*N. alba* extract (200)	10.83 ± 1.22^b^	12.67 ± 1.58^b^	71.83 ± 10.36^b^	107.7 ± 10.14^c^	46.38 ± 3.32^c^	39.10 ± 2.98^c^

Values represent mean ± SEM (n = 6);

a *P* < 0.05;

b *P* < 0.01;

c *P* < 0.001 vs.

vehicle-treated control group (one-way ANOVA followed by Dunnett’s “t” test); % OAE = percentage of open arm entries; %TSOA (sec) = percentage of total time spent in open arm in seconds

#### Open Field Test

In the OFT, diazepam treated mice showed significant increase (*P* < 0.05) in the number of rearings, number of squares crossed and number of assisted rearings during 5 min interval of test as compared to vehicle treated groups. *N. alba* extract treated rats (100 and 200 mg/kg) also produced significant increase in the number of rearings (*P* < 0.05), number of assisted rearings and number of squares crossed (*P* < 0.01) [Table 2].

**Table 2:** Effect of *N. alba* extract on behavior of mice in open field test

*Treatment (mg/kg, p.o.)*	*No. of rearings*	*No. of assisted rearings*	*No. of squares crossed*
Vehicle	10.17 ± 1.77	21.00 ± 3.36	118.2 ± 6.57
Diazepam (1)	24.00 ± 2.12^a^	37.00 ± 3.90^a^	187.5 ± 21.22^a^
*N. alba* extract (100)	25.5 ± 4.47^a^	40.67 ± 4.42^b^	209.2 ± 24.73^b^
*N. alba* extract (200)	24.17 ± 4.05^a^	38.33 ± 4.28^a^	232 ± 18.05^b^

Values represent mean ± SEM (n = 6);

a *P* < 0.05;

b *P* < 0.01; vs.

vehicle-treated control group (one-way ANOVA followed by Dunnett’s “t” test)

#### Light and Dark Box

Treatment with diazepam significantly increased the time spent (*P* < 0.001) in light box as well as the number of crossings (*P* < 0.05) between the light and dark boxes, whereas the time spent in dark box (*P* < 0.001) and duration of immobility (*P* < 0.01) were significantly reduced. *N. alba* treated mice also showed significant increase (*P* < 0.001) in the time spent in light box and the number of crossings between light and dark boxes. However, the time spent in dark box (*P* < 0.01) and duration of immobility were significantly reduced (*P* < 0.05) as compared to the vehicle treated group [Table 3].

**Table 3:** Effect of *N. alba* extract on behavior of mice in light and dark test

*Treatment (mg/kg, p.o.)*	*Time spent in lighted box (sec)*	*Time spent in dark box (sec)*	*No. of crossings*	*Duration of immobility (sec)*
Vehicle	119.2 ± 12.83	209.0 ± 19.93	16.83 ± 1.53	37.17 ± 3.74
Diazepam (1)	198.2 ± 12.64^c^	117.8 ± 10.42^c^	26.50 ± 3.212^a^	21.00 ± 2.95^b^
*N. alba* extract (100)	229.8 ± 16.39^c^	139.2 ± 8.57^b^	28.67 ± 2.71^b^	23.67 ± 1.83^a^
*N. alba* extract (200)	253.0 ± 7.34^c^	144.5 ± 4.68^b^	27.50 ± 1.31^a^	26.17 ± 3.26^a^

Values represent mean ± SEM (n = 6);

a *P* < 0.05;

b *P* < 0.01;

c *P* < 0.001 vs.

vehicle-treated control group (one-way ANOVA followed by Dunnett’s “t” test)

#### Foot Shock Induced Aggression Test

The delivery of 60-Hz foot shock current to a vehicle treated group showed aggressiveness in terms of increase in number of vocalization, number of leaps, number of rearing, number of biting/attacks and number of times they face each other. Diazepam treatment inhibited aggressiveness induced by foot shock current as indicated by a significant decrease in number of vocalization and rearing and also showed significant reduction (*P* < 0.01) in number of biting/attacks and facing each other.

Treatment with *N. alba* extract (100 and 200 mg/kg) significantly (*P* < 0.01) inhibited aggressiveness induced by foot shock current as evidenced by reduction in number of vocalization, rearing, biting/attacks, leaps and facing each other [Table 4].

**Table 4:** Effect of *N. alba* extract on behavior of mice in foot shock induced aggression test

*Treatment (mg/kg, p.o.)*	*Behavior responses*				
	*Vocalization*	*Leaping*	*Rearing*	*Biting/attack*	*Facing each other*
Vehicle	177.7 ± 32.06	32.33 ± 7.31	35.67 ± 1.45	14.00 ± 1.15	18.33 ± 2.02
Diazepam (1)	92.67 ± 10.73^a^	—	13.00 ± 3.60^a^	1.66 ± 1.66^b^	9.00 ± 1.73^b^
*N. alba* extract (100)	86.67 ± 7.68^a^	3.66 ± 2.72^b^	15.33 ± 5.36^a^	3.66 ± 2.72^a^	8.33 ± 0.88^b^
*N. alba* extract (200)	81.00 ± 5.19^a^	—	12.67 ± 5.23^a^	4.00 ± 2.30^a^	10.33 ± 0.88^a^

Values represent mean ± SEM (n = 6);

a *P* < 0.05;

b *P* < 0.01; vs.

vehicle-treated control group (one-way ANOVA followed by Dunnett’s “t” test)

#### Rota Rod Test

In this test, *N. alba* (100 and 200 mg/kg) significantly reduced the time spent by the animals on revolving rod when compared to control (*P* < 0.05). The standard drug diazepam also showed significant effect when compared to control (*P* < 0.01). Low dose of drug (100 mg/kg) did not show any significant effect at 30 and 45 min time intervals [Table 5].

**Table 5:** Effect of *N. alba* extract in mice on rota rod performance

*Treatment (mg/kg, p. o.)*	*Time (sec) of animals remained without falling from revolving rod*					
	*15*	*30*	*45*	*60*	*75*	*90 min*
Vehicle	262.2 ± 12.03	237.0 ± 9.67	218.0 ± 7.92	188.2 ± 4.96	165.5 ± 3.68	140.7 ± 6.52
Diazepam (1)	248.3 ± 11.78	203.2 ± 6.17	165.3 ± 9.50^b^	118.5 ± 11.98^c^	93.00 ± 11.07^c^	84.30±7.78^c^
*N. alba* extract (100)	270.2 ± 12.97	225.7 ± 13.34	186.5 ± 10.12	141.0 ± 7.74^b^	116.5 ± 5.57^c^	91.67 ± 5.70^c^
*N. alba* extract (200)	256.3 ± 14.75	189.2 ± 13.97^a^	145.8 ± 9.50^c^	86.00 ± 6.37^c^	67.00 ± 4.91^c^	56.67 ± 3.33^c^

Values represent mean ± SEM (n = 6);

a *P* < 0.05;

b *P* < 0.01;

c *P* < 0.001 vs.

vehicle-treated control group (one-way ANOVA followed by Dunnett’s “t” test)

## Discussion

Anxiety, like all emotions, has cognitive, neurobiological and behavioral components. It is a negative emotion that occurs in response to perceived threats that can come from internal or external sources and can be real or imagined.[18] The incidence of anxiety in the community is very high and associated with lot of morbidity.[19] Ethnomedical and pharmacological knowledge about the plant under study would allow us to evaluate central nervous system activity, which could be used to treat anxiety type of disorders. The present work has shown that anxiolytic activity by the *N. alba*, as assessed by OFT, light/dark box, EPMT, FSIAT and motor coordination test.

The EPMT is used to evaluate psychomotor performance and emotional aspects of rodents. Results obtained on the elevated plus maze after treatment with *N. alba* (100 and 200 mg/kg) revealed anxiolytic activity, since increases in open arm entry parameters are the most representative indices of anxiolytic activity.[20] Time spent on the central platform appears to be related to decision making and/or risk assessment, and the total arm entries is a contaminated measure reflecting changes in anxiety or in general activity.[21]

The anxiolytic-like activity was also observed in the light/dark box. L and DT is an ethological-based approach-avoidance conflict test and it is sensitive to drugs that affect anxiety. In this test, the number of transitions between the light and dark compartments as well as the time spent in the light side are recognized as anxiety indices, despite the transition parameter being highly dependent on locomotor activity.[22] Mice treated with *N. alba* (100 and 200 mg/kg) showed increase in the time spent in the light compartment and no changes in the numbers of shuttle crossings, confirming the activity upon the main anxiolytic parameter. The observed anxiolytic effect of *N. alba* may be due to the agonistic effect on GABA/benzodiazepine receptor complex, or antagonize the 5-HT1B receptor or agonize the 5-HT1A receptor.[23,24]

The OFT is used to evaluate the animal emotional state. The open field model examines anxiety related behavior characterized by the normal aversion of the animal to an open, brightly lit area. Thus, animals removed from their acclimatized cage and placed in environment express anxiety and fear, by showing alteration in all or some parameters. Anxiolytic treatments reduce such fearful behavior of animals in open field.[25] Statistical analysis of the data obtained from these experiments supported anxiolytic-like activity of *N. alba* extract at both the doses (100 and 200 mg/kg) as its effect shows significant increase in the number of rearings, number of assisted rearings and number of squares crossed, as compared to the vehicle treated group, which indicates its anxiolytic-like effect.

FSIAT is based on the exposure of an animal to a threatening situation which produces a behavior referred to as aggression. This method provides an assessment of the animal’s level of defensive behavior, which reflects the extent of uneasy or fearful experiences. The agents that minimize these experiences would indicate the anxiolytic property. Analysis of data of this study has revealed that *N. alba* extract significantly inhibited aggression behavior induced by foot shock, indicating that it has potential anxiolytic property. The serotoninergic system is implicated in aggressive states and it has been hypothesized that decreasing serotoninergic activity may encourage aggressive behavior.[26] Since both anti-anxiety and anti-aggressive effects are seen with 5-HT1A antagonists, it is assumed that *N. alba* may also interact with the 5-HT1A receptors.

RRT was first introduced to screen the assay of neurotoxicity of anticonvulsants and later was reported to predict motor dysfunction produced by centrally acting drugs to determine possible alterations in the motor coordination ability of the animal, often caused by the use of sedative and antipsychotic drugs.[17] In this test, the difference in the fall of time from the rotating rod between the vehicle and extract treated groups is taken as an index of muscle relaxation. The skeletal muscle relaxation together with taming or calming effect, also reduces anxiety and tension. Thus, in this study, both the doses of *N. alba* extract (100 and 200 mg/kg) and diazepam significantly reduced the fall of time of the mice from the rotating rod, indicating the skeletal muscle relaxant activity.

Earlier reports on the chemical constituents of plants and their pharmacology suggest that plants containing flavonoids, alkaloids, phenolic acids, essential oil, saponins and tannins possess activity against many CNS disorders.[27] A survey of the literature on *N. alba* revealed the presence of tannic acid, gallic acid, alkaloids, sterols, flavonoids, glycosides, hydrolyzable tannins and high–molecular-weight polyphenolic compounds. It is possible that the mechanism of anxiolytic action of *N. alba* could be mediated by synergistic action of these phytochemicals.

The results obtained in this study suggest that the ethanolic extract of *N. alba* possesses anxiolytic and muscle relaxant properties. Thus, *N. alba* has potential clinical applications in the management of anxiety and muscle tension disorders. Further investigations are warranted for elucidating the exact mechanism and bioactive compounds.
